# Electronic prescription systems in Greece: a large-scale survey of healthcare professionals’ perceptions

**DOI:** 10.1186/s13690-024-01304-6

**Published:** 2024-05-10

**Authors:** Margarita Grammatikopoulou, Ioulietta Lazarou, George Giannios, Christina Asimina Kakalou, Martha Zachariadou, Maria Zande, Haralampos Karanikas, Eleftherios Thireos, Thanos G. Stavropoulos, Pantelis Natsiavas, Spiros Nikolopoulos, Ioannis Kompatsiaris

**Affiliations:** 1 Information Technologies Institute, Centre for Research and Technology Hellas, Thessaloniki, Greece; 2 Institute of Applied Biosciences, Centre for Research and Technology Hellas, Thessaloniki, Greece; 3 Ergobyte SA, Thessaloniki, Greece; 4 Department of Computer Science and Biomedical Informatics, University of Thessaly, Lamia, Greece

**Keywords:** Electronic prescribing, Healthcare professionals, System evaluation, End-user requirements, Electronic prescription system, Healthcare systems, Survey

## Abstract

**Background:**

The national e-prescription system in Greece is one of the most important achievements in the e-health sector. Healthcare professionals’ feedback is essential to ensure the introduced system tends to their needs and reduces their everyday workload. The number of surveys collecting the users’ views is limited, while the existing studies include only a small number of participants.

**Methods:**

In this study, healthcare professionals’ perceptions on e-prescription are explored. For this, a questionnaire was distributed online, containing closed- and open-ended questions aiming to address strengths and identify drawbacks in e-prescription. Answers were collected from primary health care physicians, specialized medical doctors and pharmacists.

**Results:**

In total, 430 answers were collected (129 from primary health care physicians, 164 responses from specialized medical doctors and 137 pharmacists). Analysis of the collected answers reveals that the views of the three groups of healthcare professionals mostly converge. The positive impact e-prescribing systems have on the overall prescribing procedure in preventing errors and providing automation is commented. Among gaps identified and proposed improvements, health care professionals note the need for access to information on adverse drug reactions, side effects, drug-to-drug interactions and allergies. Flexible interaction with Therapeutic Prescription Protocols is desired to ameliorate monitoring and decision-making, while drug dosing features, and simplified procedures for copying, repeating, canceling a prescription, are perceived as useful to incorporate.

**Conclusions:**

Collecting healthcare professionals’ feedback is important, as their views can be transcribed to system requirements, to further promote e-prescribing and improve the provided health care services by facilitating decision making through safer and more efficient e-prescription. Introduction of the identified improvements can simplify the everyday workflow of healthcare professionals. To the best of our knowledge, a survey with more than 400 answered questionnaires on the use of e-prescription systems by healthcare professionals has never been conducted in Greece before.

**Supplementary Information:**

The online version contains supplementary material available at 10.1186/s13690-024-01304-6.

**Table:** 

Text box. 1 Contributions to the literature
• This research adds to the existing literature as it reinforces the need for user-centered design in healthcare technology.
• It highlights the direct link between user involvement and the development of digital clinical tools that tend to clinicians’ needs and address challenges faced in their everyday clinical practice.
• There is limited research on clinicians’ perceptions on the national e-prescribing system in Greece, implemented more than ten years ago, regarding its usability, strengths and weaknesses.

## Introduction

Electronic Prescription (e-prescription) systems and Computerized Physician Order Entry (CPOE) systems are increasingly being adopted by healthcare organizations [1, 2]. In some countries, like Greece, there is a national e-prescription system aiming to facilitate healthcare professionals (HCPs) in documenting transactions, prevent malicious or fraudulent medicine prescription and also help in the assessment/retain of healthcare costs. The focus of such systems up to now has been given mainly to administrative and auditing aspects of healthcare delivery [3, 4]. Lately there is a trend to complement and advance their functionality with features aiming to reinforce quality and safety of care. Since these systems typically aim to prevent potential Adverse Drug Reactions via some kind of “alert” raising, the user experience is identified as a crucial part of the overall system quality and needs to be evaluated in a systematic manner [5].

In Greece, the swift introduction of the national e-prescription system in 2011 was aiming mainly to restrict pharmaceutical expenditure, to improve the collaboration between doctor and pharmacy, enhance patient safety and to promote evidence-based policy development through the collected data [6]. The development started in 2010 and a pilot run in October of the same year, while the official launch was in January 2011. By the fall of 2013 prescriptions were almost fully covered as 98% of pharmacists and 90% of doctors were prescribing electronically [6, 7]. Further alterations and updates were made to improve functionalities and simplify features, while in 2013 the order of lab tests via the same e-prescription user interface (UI) was introduced. The introduction of e-prescription in Greece is presented in detail in the work of [6]. It is discussed how the e-prescription system was built by leveraging on the advantages of the existing infrastructure and by making continuous adaptations to avoid preserving the infrastructure’s weaknesses. In the work of Pangalos the popularity of the system is commented [8].

There is limited research, regarding the Greek e-prescription platform, concerning mainly expenditure and auditing aspects of the introduced system [3, 9]. Surveys discussing the acceptability of the system, assessing the opinions of key stakeholders like HCPs (doctors, pharmacists) and patients, whose feedback and input are valuable to evolve healthcare services are limited, while they include only a small number of participants [10–12].

In detail, in the work of Minarikova (2015) a survey including 29 pharmacists, 11 doctors and 56 patients is presented [10]. The participants were asked to complete a questionnaire assessing ease of use, safety, level of service, administrative and back-office workload, economic and technological aspects of e-prescription in Greece. The three groups acknowledge that the introduced e-prescribing system greatly promotes medication safety and can increase the communication time between the patient and the doctor or pharmacist. The groups comment that healthcare costs concerning public expenditure have been limited. Room for improvement exists regarding the system itself, while training of the HCPs is viewed as important for them to benefit from the advantages e-prescribing has to offer.

A more recent study was conducted with the participation of 55 physicians, 13 nurses and 9 administrative personnel of the Primary Health Care sector in two regions in Greece [11]. Participants were asked to evaluate the usefulness, ease of use, ease of learning and user satisfaction regarding e-prescribing and e-appointment systems. Users’ satisfaction received a lower score compared to the other categories with the authors noting that the reasons behind that revealed trend need to be further investigated.

Furthermore, the study of Nikou included 157 HCPs (80 pharmacists and 77 physicians) [12]. Reported findings demonstrate that even though participants perceive the systems as reliable and useful, the system’s “ease of use” aspect was rated low .

### Study aim

The present study aims to collect HCPs’ perceptions on the Greek national e-prescription system provided by IDIKA^1^^,^^2^ (e-government centre for social security services). The end users’ feedback is important as it can be converted to technical requirements and components to be incorporated in a system, elevate its functionality and reinforce quality and safety of use. In this context, the PrescIT project^3^ aims to develop a platform to facilitate e-prescribing and support clinical decision-making in terms of preventing potential Adverse Drug Reactions (ADRs) and/or Drug-Drug Interactions (DDIs). With emphasis on the end-users we aim to develop a system that tends to their needs and promotes efficient and safe e-prescribing.

For the purpose of this study, primary health care physicians (PHCPs), specialized medical doctors (SMDs) and pharmacists were addressed and asked for their opinion via a self-reported online distributed questionnaire survey conducted in the context of the PrescIT project. The survey examines overall ease of use, user satisfaction but also focuses on identifying missing features and proposed improvements. To the best of our knowledge, a survey with more than 400 answered questionnaires on the use of e-prescription systems by HCPs has never been conducted in Greece before.

## Materials and methods

### Participants and settings

The questionnaires were distributed online via the Google Forms platform through mailing lists to respective HCPs in clinics, hospitals, healthcare facilities, associations, accompanied by a brief description of the project and its aims, information on data collection and informed consent. The period for distributing / circulating the questionnaires and collecting answers was from 31/08/2021 to 07/03/2022.

In total, 430 responses were collected, namely 129 from PHCPs, 164 responses from SMDs, while 137 pharmacists also participated in the survey. According to the Hellenic Statistical Authority’s^4^ report from December 2022, the total number of HCPs (comprising doctors and pharmacists) reached 77,918 for the year 2021. Sample size calculation [13] determined that a minimum of 383 participants should be included to ensure a confidence level of 95% with a margin of error of ± 5%. For this, the 430 responses were considered a viable survey sample to report results. All questionnaires collected were considered valid and could be included in the analysis, as they were complete (open-ended questions were not mandatory for the completion) and participants stated that they had experience with the national e-prescription system (this was considered as a prerequisite). Demographics of the participants regarding their age, gender and work experience are presented in Table 1. The majority of PHCPs and SMDs responded that they hold “6–10 years” of experience with e-prescribing systems (72.1% and 72.6% respectively), while 51.1% of pharmacists reported “11 + years” of experience with the answer “6–10 years” following with 38.7% (Table 1).

**Table 1:** Demographic characteristic of the participants per group

	Primary Health Care Physicians		Specialized Medical Doctors		Pharmacists	
	*N* = 129	%	*N* = 164	%	*N* = 137	%
**Gender**						
Female	38	*29.4*	53	*32.3*	71	*51.8*
Male	89	*69.0*	110	*67.1*	66	*48.2*
Prefer not to say	2	*1.6*	1	*0.6*	-	*-*
**Age**						
20–29	1	*0.8*	-	*-*	5	*3.6*
30–39	4	*3.1*	17	*10.4*	36	*26.3*
40–49	44	*34.1*	50	*30.5*	48	*35.0*
50–59	48	*37.2*	61	*37.2*	31	*22.6*
60–69	28	*21.7*	33	*20.1*	15	*11.0*
70+	4	*3.1*	3	*1.8*	2	*1.5*
**Work experience (given in years)**						
1–10	42	*32.6*	70	*42.7*	46	*33.6*
11–20	47	*36.4*	48	*29.3*	47	*34.3*
21–30	24	*18.6*	34	*20.7*	23	*16.8*
31–40	16	*12.4*	12	*7.3*	21	*15.3*
**Experience using e-prescription systems (given in years)**						
> 0–5	8	*6.2*	25	*15.2*	14	*10.2*
6–10	93	*72.1*	119	*72.6*	53	*38.7*
11–15	24	*18.6*	18	*11.0*	53	*38.7*
16+	4	*3.1*	2	*1.2*	17	*12.4*

### Research instrument – questionnaires on e-prescription (ePreQs)

The questionnaire items are based on the published questionnaire of Kauppinen [14], used to assess the impact of e-prescriptions on the medicine dispensing process in Finnish community pharmacies, and further existing literature [15, 16]. The questionnaire items were shaped to our research’s needs, while additional questions were identified through research and available studies aiming to assess and evaluate e-prescribing systems in Greece [10–12]. The developed Questionnaires on e-prescription (ePreQs) were internally reviewed by the PrescIT project’s team for their completeness and suitability. Comments and feedback were requested from doctors of the consortium and changes were made where appropriate so that the phrasing was comprehensive and in accordance with the medical terminology. Each questionnaire was intended for a separate category of HCPs (PHCPs, SMDs, pharmacists).

The ePreQs comprise closed- and open-ended questions. Introductory questions aim to gather information on participants’ experience and familiarity with e-prescribing and the handled volume of prescriptions. Ease of use and benefits of e-prescription systems are assessed, while potential issues are detected along with desirable features and suggested improvements. The purpose of open-ended questions is to identify end-user needs through the flexible and restriction free recording of answers, while closed-ended questions (use of Likert scale items with answer options ranging from 1 = Strongly disagree to 5 = Strongly agree) facilitate quantitative analysis. The questionnaires include common questions and items allowing comparisons between the different groups and also individual / separate questions taking into consideration the unique aspects of the work of the HCPs. For example, focus to prescription execution is given for pharmacists, while evaluation of clinical tools (e.g., Therapeutic Prescription Protocols, TPPs) is contained in the questionnaires addressing the HCPs. The time required to complete the questionnaires was approximately 10–15 min. The ePreQs are available on the project’s website^5^ (in Greek) and can be found in the Supplementary Material (in English).

### Statistical analysis

Data analysis was performed using the SPSS software Version 25.0^6^. Descriptive statistics were used to assess HCPs’ responses as percentage (%) of the participants, with line charts revealing trends in participants’ answers.

## Results

### Frequency of e-prescribing and systems used

**Fig. 1:**
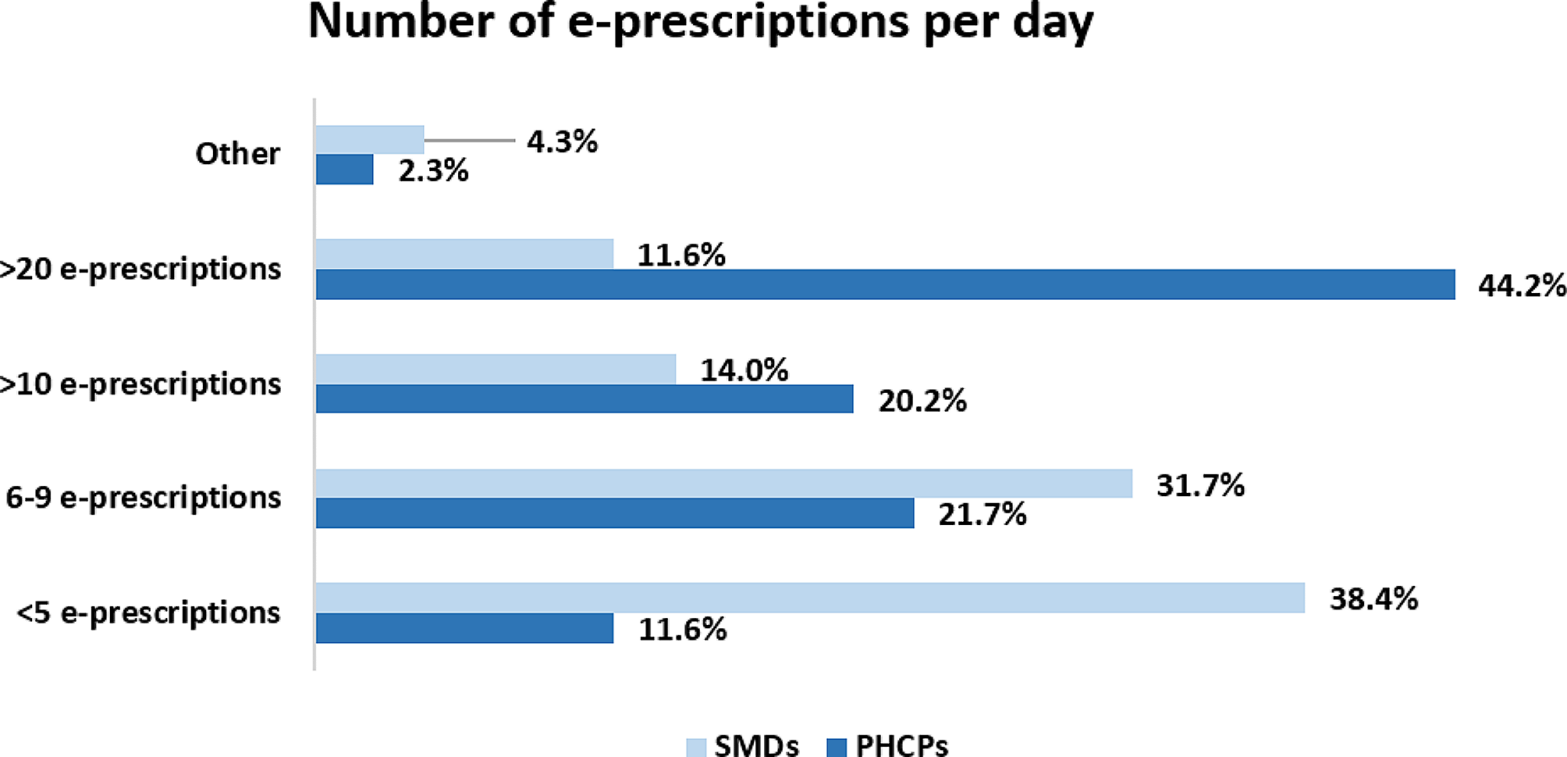
Number of e-prescriptions per day administered by PHCPs and SMDs

The vast majority of PHCPs (94.7%) is prescribing medications daily and 44.2% of them are registering more than 20 electronic prescriptions per day (Fig. 1). In addition, 76.8% of SMDs prescribe daily, with 38.4% of them stating that they prescribe < 5 e-prescriptions per day and 31.7% of them 6–9 prescriptions per day (Fig. 1). 94.2% of pharmacists execute prescriptions daily, while they almost unanimously (91.2%) stated that the percentage of prescriptions processed through an e-prescribing system on a daily basis is greater than 75%.

The UI provided by the Greek national e-prescription system operated by IDIKA is used by 58.9% of PHCPs with the rest of the participants using third party applications (which still use the IDIKA system’s back-end via the respective Application Programming Interface - API). The vast majority of SMDs use the UI provided by IDIKA (91.5%), while among pharmacists, the IDIKA system holds second place along with a third party application.

### Impact of e-prescription

Regarding the open-ended question “How has e-prescribing affected your work?“, the answers collected showed a positive connotation while an equal number of answers revealed a negative impact on the work of individuals. It was observed that even though PHCPs and SMDs recognize the positive effects of e-prescribing they also identify problems and malfunctions of the systems. Both groups noted that the procedure is easier, it allows to follow the patients’ medical history (via the patients’ electronic health record, EHR) and increases the time available to interact with the patient. Notably, at the same time, under the negative aspects, they mentioned that in case of system malfunction they are unable to proceed with the prescription, with the delay causing stress and reducing the interaction time with the patient. For 88.3% of pharmacists, e-prescribing has had a positive effect on their work routine. Open-ended answers gathered, note that it has increased prescription processing speed and efficiency. Furthermore, ease of use and avoiding errors are pointed out.

**Fig. 2:**
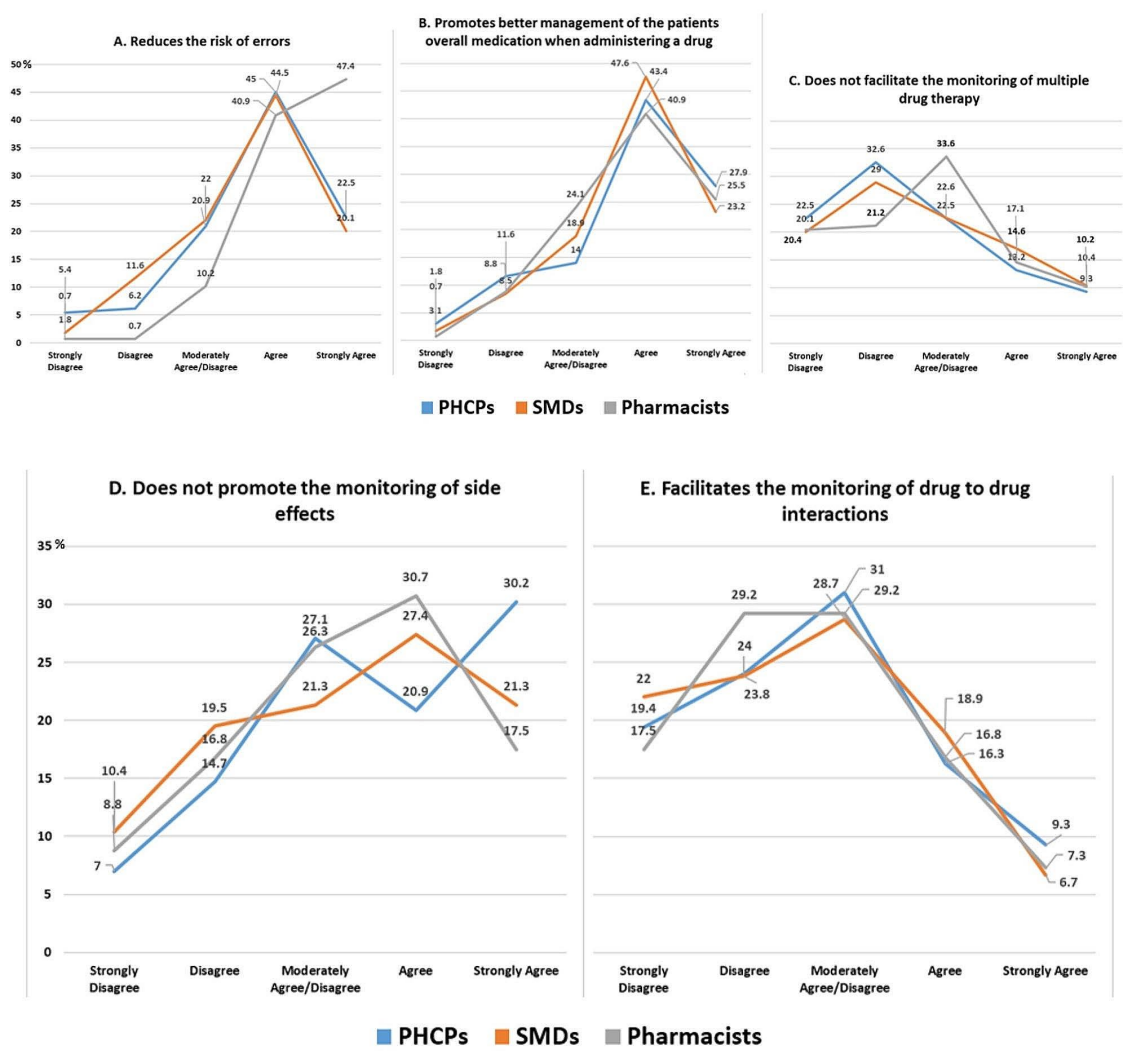
Line charts depicting participants’ answers per group on e-prescribing

Additionally, participants were asked to comment on a set of sentences regarding e-prescription. All three groups agreed that e-prescription reduces the risk of errors (67.5% primary care physicians, 64.6% SMDs and 88.3% pharmacists answered “Agree” or “Strongly Agree”, Fig. 2A). Participants perceived that e-prescription offers potential for improved management of patients’ overall medication (71.3% PHCPs, 70.8% SMDs and 66.4% pharmacists answered “Agree” or “Strongly Agree”, Fig. 2B). Moreover, responses showed that participants believe in some extent that multiple drug therapy can be monitored through e-prescription [55.1% PHCPs, 49.1% and 41.6% pharmacists answered “Strongly Disagree” or “Disagree”, (**Figure C**) (this statement was provided as a reverse-polarity question)]. However, pharmacists’ highest percentage (33.6%) was collected for the answer “Moderately Agree/Disagree” regarding the statement “E-prescription does not facilitate the monitoring of multiple drug therapy”. Regarding the monitoring of side effects and drug to drug interactions, approximately half of the participants in each group responded that this is not promoted and facilitated by e-prescription [51.1% PHCPs, 48.7% SMDs, 48.2% pharmacist answered with “Agree” or “Strongly Agree” (Fig. 2D) and 43.4% % PHCPs, 45.8% SMDs, 46.7% pharmacist answered with “Disagree” or “Strongly Disagree” correspondingly (Fig. 2E) (the statement was provided as a reverse-polarity question)]. However, it is also noted that 27.1% PHCPs, 21.3% SMDs and 26.3% of the pharmacists responded with “Moderate Agree/Disagree” with the statement “E-prescription does not promote the monitoring of side effects” (Fig. 2D). This answer gathered even higher percentages for the statement “E-prescription facilitates the monitoring of drug to drug interactions” (31% PHCPs, 28.7% SMDs and 29.2% of the pharmacists) (Fig. 2E).

Detailed examination of the impact that e-prescription has on different phases of prescription, followed. Thirteen phases related to prescribing were presented (Table A, Supplementary Material) and participants were asked to comment on whether they thought e-prescriptions had affected each of them. The highest percentages of “Strongly agree” and “Agree” responses (in total > 70%) were given by all three groups for the sentences: [e-Prescription has facilitated] Recipe registration (compared to handwritten prescriptions), Recipe information check, Monitoring of intervals of execution of the prescription from the moment of prescribing. In addition, > 80% of pharmacists responded “Strongly agree” and “Agree” at the following: [e-Prescription has facilitated] Checking and completing the participation rate (cost sharing rate), Transfer prescription data to the pharmacy data system.

Afterwards, participants were given a series of questions specifically assessing the national e-prescription UI.

**Fig. 3:**
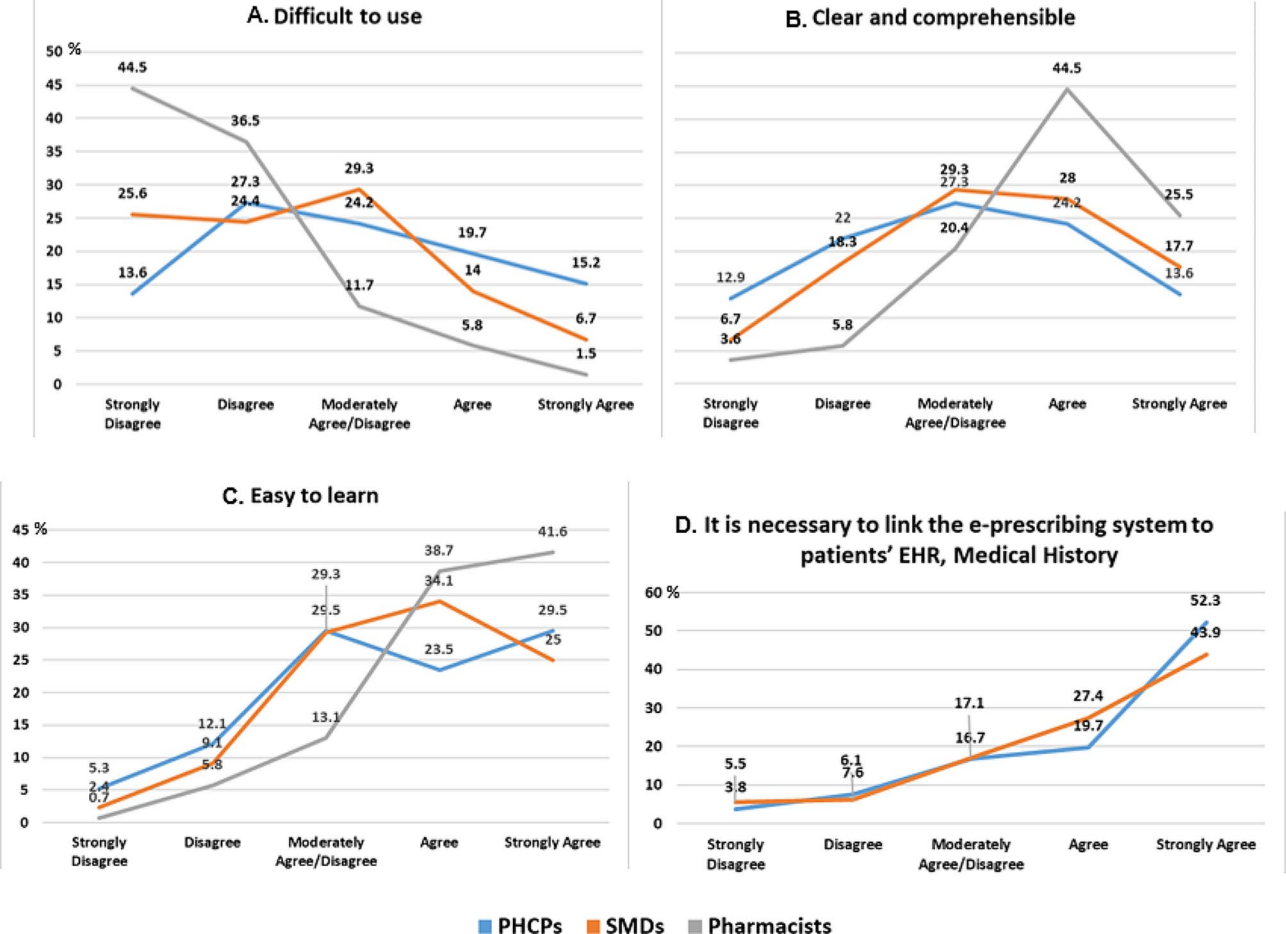
Participants’ answers regarding the IDIKA system given in line charts

Regarding the national e-prescription system, pharmacists’ answers appeared to be the most undivided, with 81% finding it not difficult to use (Fig. 3A), 70% perceiving the system as clear and comprehensible (Fig. 3B) and 80.3% responding that it is easy to learn (Fig. 3C).

Within the PHCPs and the SMDs groups, answers were more varied (Fig. 3A-3C). In detail, PHCPs answers were divided when asked to respond to the statement “The national e-prescription system is difficult to use” (13.6% “Strongly Disagree”, 27.3% “Disagree”, 24.2% Moderately Agree/Disagree”, 19.7% “Agree” and 15.2% “Strongly Agree”) (Fig. 3A). For SMDs, while 50% responded with “Strongly Disagree” or “Disagree”, 24.2% answered with “Moderately Agree/Disagree” to the same statement (Fig. 2A). Moreover, when asked to respond to the statement “The national e-prescription system is clear and comprehensive”, although answers vary within groups it is noted that the responses from PHCPs and SMDs exhibit similarities (Fig. 3B). Additionally, in Fig. 3C, it can be observed that even though 53% PHCPs and 59.1% SMDs perceive the system as easy to learn, 29.5% and 29.3% answered with “Moderately Agree/Disagree”.

The national e-prescription system currently is the main data provider to the patients’ national EHR, as it provides the dispensed prescriptions along with the associated diagnosis. Participants’ answers reflect the importance of having access to this information, with 72% of the PHCPs and 71.3% of the SMDs finding it necessary to link the e-prescription system to patient’s EHR and medical history (Fig. 3D).

**Fig. 4:**
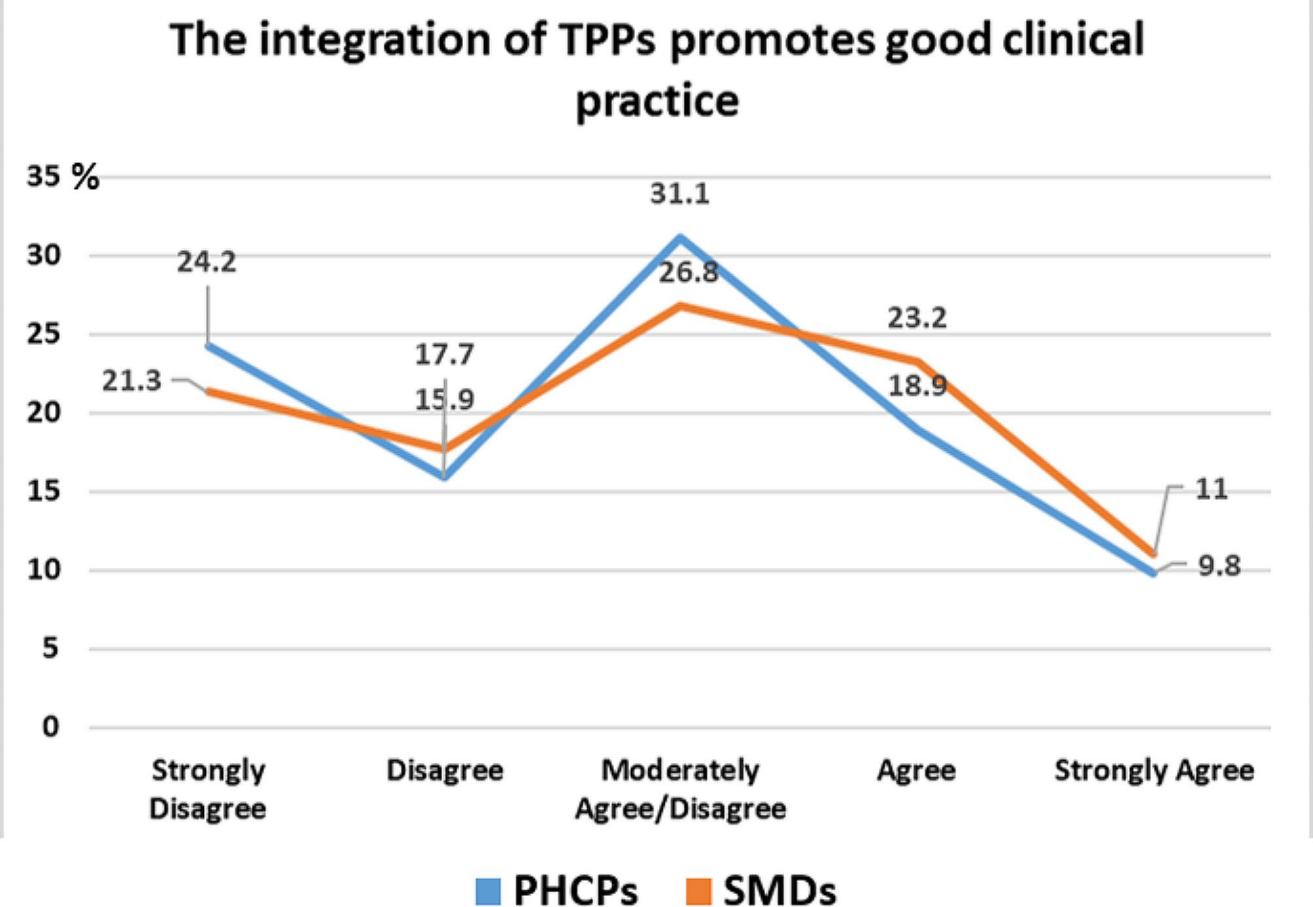
Participants’ answers regarding TPPs implemented in the IDIKA system

Regarding the implementation of the integrated Therapeutic Prescription Protocols (TPPs), the answers of the doctors are divided, and no clear tendency can be detected as to whether they consider them to promote good clinical practice (Fig. 4). Furthermore, this lack of a trend could be also observed in the next questions, as 40% of the participants perceive the TPPs as easy to learn, while 50% of the participants in the next question note that the TPPs are not easy to use.

### Privacy

Even though 80% of all three groups considered the system to be secure, the rest of the participants were asked to identify which issues they consider problematic. Participants mainly commented on the ease of access by third parties knowing the patient’s social security number (AMKA), thus being able to access patient’s medical history. In addition, access to the system is feasible, as passwords are inevitably known among employees. For this, they propose the introduction and regular update of a security question.

### Perceived clarity of regulations

When asked for the participants’ opinion regarding clarity of the official regulations on e-prescribing, approximately 60% of all HCPs replied that they are clear. The rest of the participants identified as issues the lack of information (some were even unaware of the existence of regulations), along with ambiguities in directives and missing explanations.

### Benefits regarding e-prescribing

The benefits listed by physicians in the open-ended questions include error prevention, gained control and transparency of the prescribing process. Monitoring the overall treatment of the patient and access to the medical history were also mentioned as main benefits. Answers often referred to the ease and speed of the prescribing process using an e-prescription system compared to handwritten prescriptions. Physicians prescribing using a platform other than IDIKA unanimously answered that the respective system used is faster and easier, the renewal of the prescriptions is uncomplicated while they consider the use of the TPPs as facile. The most common answer given by the participating pharmacists is the safety offered by e-prescription, due to the reduction of errors when executing a prescription (errors mentioned varying from illegible handwritten prescriptions to giving the wrong medication to the patient). Also, the speed and time saving through the system are recognized. Reference was made on how over-prescribing and fake prescriptions are avoided, while emphasis was given again to the patient’s medical history. Pharmacists also mentioned the convenience e-prescription offers in terms of administrative matters like checking patient’s participation rates and drug prices.

### Identified issues and proposed improvements

Participants identified the lack of reference to patients’ allergies (45%), the incorrect pharmaceutical form of the medication (ml, mg, tabs, etc.) (32.6%), as well as the lack of reporting on adverse drug reactions (31%) as more often accounted problems creating uncertainties. Other obstacles seem to be the wrong total amount of medicine in the package, the lack of dosage information or the vague or incorrect dosing instructions and the lack of useful information as for example the patients weight. Pharmacists ranked the unclear or incorrect dosing instructions as the most important issue (64.2%), the lack of reference to patient’s allergies (38.7%) and the lack of reporting adverse drug reactions (34.3%). The list of potential gaps / identified issues can be found in Table 2 along with participants’ collected answers.

**Table 2:** List of potential gaps

		HCPs (%)	Pharmacists (%)
1.	Incorrect medicinal product	27.1	21.2
2.	No information on adverse drug reactions	31.0	34.3
3.	Incorrect pharmaceutical form (ml, mg, tabs, etc.)	32.6	25.5
4.	Incorrect total amount of medicine contained	23.3	32.8
5.	Unclear or incorrect dosage instructions	27.1	64.2
6.	Missing dosage instructions	25.6	28.5
7.	Useful information is missing (e.g., the patient’s weight)	26.4	13.1
8	No information on patients’ allergies	45.0	38.7
9.	Specific dosage instructions or specific purpose of use missing	25.6	21.2
10.	Other	10.9	13.1

Twenty possible improvements of the e-prescription system were presented to the participants and they were asked to select at least three they considered important. Table 3 shows the four selected improvements from each group that gathered the highest percentages. All three groups perceived as crucial the “Link/Connection with patients’ EHR, Medical History, Diagnoses, Comorbidities, Allergies”. For the group of pharmacists and SMDs the second preferably suggested improvement is information on drug interactions. For PHCPs, “Integration of TPPs allowing flexible interaction” is in second place (fourth in preference for SMDs), followed by “Easy / simple drug selection” and “Reduced actions (e.g., mouse clicks)”. The third preferred improvement for pharmacists is the “Communication between the prescribing clinician and the pharmacy for the availability of a specific drug” and next is the “Warning for prescribing high doses (not in accordance with the drug’s SPC)”. The third selected improvement for the SMDs and pharmacists was the “Easy / simple drug selection”. It is noted that for SMDs, further improvements (Q11 – option to save favorites, Q20 – issuing reports, e.g., list of medicines prescribed to a patient, 12, 15) gathered a percentage higher than 40%.

**Table 3:** Ranking of the proposed improvements perceived as important by each group

		1st	2nd	3^d^	4th
PHCPs		Q2 (75.2%)	Q3 (53.5%)	Q1 (50.4%)	Q12 (49.6%)
SMDs		Q2 (75.0%)	Q14 (57.3%)	Q1 (45.7%)	Q3 (44.5%)*
Pharmacists		Q2 (86.9%)	Q14 (65.7%)	Q4 (61.3%)	Q15 (59.1%)
**Potential improvements**					
Q1	Easy / simple drug selection				
Q2	Link to patients’ EHR, Medical History, Diagnoses, Comorbidities, Allergies				
Q3	Integration of TPPs allowing flexible interaction				
Q4	Communication between the prescribing clinician and the pharmacy for the availability of a specific drug				
Q12	Reduced number of actions (mouse clicks)				
Q14	Information on drug interactions				
Q15	Warning for prescribing high doses (not in accordance with the drug’s SPC)				

Additionally, in the open-ended questions, most participants pointed out technical problems such as delays, frequent connection interruptions, slow response speed. Extended references were made regarding the integrated TPPs. The participants seek more flexibility in management and the overall interaction with the TPPs. Specific features were identified that could improve the TPPs’ usability, for example, the ”prescription repetition” function in the context of e.g., a refill request, as this is not possible prior to the completion of a TPP currently followed by a patient.

They also noted the difficulty of copying recipes and the lack of “memory” so that it is necessary to fill in again information about e.g., dosage. They consider the connection/link to the patient’s medical record necessary, while the option to upload laboratory results was also mentioned as a desirable feature. A significant number of pharmacist respondents identified problems related to drug dosing and urged for better ways to record dosage, while they often reported discrepancies between the prescribed medication amount and the proposed dosage regimen. Pharmacists also suggested the existence of restrictions for doctors when prescribing. As the main area that requires development, they identified the control of drug-to-drug interactions to avoid possible side effects. They considered it necessary to have access to the patient’s pharmacotherapy history. This exists currently at the pharmacy level but there is a need to expand the implementation to the patient level. Promoting intangible prescription, many reported the annulment of the use of paper and self-adhesive barcodes (used for auditing aspects). In addition, they noted the lack of improvements, in the form of regular updates made, to enhance the available systems and their functionalities.

## Discussion

E-prescription was implemented more than ten years ago and is an integral part of HCPs work life. Given that five million prescriptions are registered monthly in Greece [8], HCPs require an e-prescribing system that meets their needs, enables interaction and does not add to the workload, promoting quality healthcare services. For this, the views of the HCPs are considered important and their feedback valuable. The analysis of the collected answers shows that e-prescribing systems have a positive impact on the overall prescribing process, as HCPs acknowledge minimization of errors and simplified procedures in comparison to handwritten prescriptions. The automation provided by the e-prescribing procedure, seems to be perceived by HCPs as a mean to decrease medication errors. E-prescribing appears to streamline the handling of prescriptions for both doctors and pharmacists. However, HCPs’ views attest that improvements could be made to the systems to further aid and support them in decision-making while promoting quality healthcare services.

The analysis of the questionnaires shows that the answers given to the open-ended questions and the closed-ended multiple-choice questions addressing the same matters converge, establishing/revealing the areas where electronic prescription systems could benefit from improvement. Furthermore, we can observe how the three groups of HCPs have similar opinions on the various subjects addressed in the questionnaires, and overall identify the same gaps and issues (line charts showing similar trends). Due to their distinct roles in the healthcare sector, differences can also be detected in participants’ answers, for example with pharmacists noting that improvements are needed to avoid medication dosing ambiguities. The groups propose changes that appear to make the prescribing process smoother and safer, improving both their own workflow and the quality of the healthcare services provided to the patients.

Main areas identified to be further examined, transcribed to components and incorporated as features in the system developed in the framework of the PrescIT project, include providing HCPs information on adverse drug reactions, side effects, drug-to-drug interactions and allergies, in this way increasing the safety of the provided healthcare services. The link to the patient’s EHR, medical history, information on past diagnoses, comorbidities, is essential to make the e-prescribing procedure easier, faster and more automated. Furthermore, the integration and shaping of TPPs in a manner that allows flexible interaction would be an important asset of the system to facilitate monitoring and decision-making. Drug dosing control features, easy/simple drug selection, available actions related to copying, repeating, and canceling a prescription are desirable features to simplify the everyday workflow of doctors and pharmacists.

Taking into account the studies conducted to assess aspects of the national e-prescription system in Greece [10–12], the present study further supports previous findings, while the large number of participants involved strengthens the results. Consistent to [12] it was observed that pharmacists expressed more positive opinions regarding ease of use compared to the feedback received by the other groups of HCPs (e.g., Fig. 3A and C). Additionally, the similar trends in responses across the different groups of HCPs, as depicted in the line charts, are also in agreement with previous findings. However, it is important to note that the extensive questionnaire developed and utilized herein, allowed for detailed input to be collected from the participants regarding their perspectives. This level of information is novel compared to the previous studies.

## Conclusions

The research presented, aimed at collecting the views of HCPs (PHCPs, SMDs, pharmacists), on e-prescription systems in Greece. This work attempts to highlight the most important and desirable features of the e-prescription systems currently used. Although the national e-prescription system in Greece is one of the most important achievements in the e-health sector, we need to recognize the main issues / gaps identified in the existing system used today. A total of 430 HCPs participated in the present study. To the best of our knowledge, to date in Greece, no other study has been conducted examining benefits and gaps of electronic prescription with such a sample size. The views of the participants, as collected and analyzed, will be translated to end-user requirements, and finally to specifications of the developed PrescIT platform to promote safe e-prescribing, support and facilitate decision-making.

### Limitations and future work

It is noted, that a questionnaire was also designed and distributed to hospital and clinic managers, aiming to assess the administrative and auditing aspect of the procedure. The feedback is taken into consideration but extensive conclusions cannot be drawn as the small sample size (*N* = 9) does not allow statistical analysis of the answers.

The self-reported questionnaires utilized Likert scale items, for this, Likert scale bias and self-reported bias need to be taken into account. It is also noted that in the present study, as convenience sampling was used, HCPs willing to participate in the online study might be overall more receptive to technology and thus familiarized with systems and tools such as e-prescription systems.

Next steps for the project include implementation of the developed PrescIT platform in the hospitals and clinics of the consortium where it will be tested in a real-world environment. Furthermore, a survey assessing user satisfaction regarding the PrescIT platform will be conducted.

### Electronic supplementary material

Below is the link to the electronic supplementary material.

Supplementary Material 1

## Data Availability

The dataset generated and analyzed during the current study is available in the Zenodo repository (https://zenodo.org/record/8096705).

## Footnotes

1 https://www.idika.gr/.

2 https://www.e-prescription.gr/.

3 https://www.prescit.com/.

4 https://www.statistics.gr/el/statistics/pop.

5 https://www.prescit.com/questionnaires/.

6 IBM Corp. Released 2017. IBM SPSS Statistics for Windows, Version 25.0. Armonk, NY: IBM Corp.
