# Supplementary material for: Electronic prescription systems in Greece: a large-scale survey of healthcare professionals’ perceptions
Source: Arch Public Health. 2024 May 10;82:68. doi: 10.1186/s13690-024-01304-6 (PMC11088065; doi:10.1186/s13690-024-01304-6)
Supplement: Supplementary file 1 — Supplementary Material 1 [file 13690_2024_1304_MOESM1_ESM.docx]

# Supplementary Material

**Section A**

**Table A.** Exploring the impact of e-prescription in various phases of prescribing. [e-Prescription has facilitated…] (given in percentages for: a=PHCPs, b=SMDs, c=Pharmacists)

|  | **Strongly Disagree** | **Disagree** | **Moderately Agree / Disagree** | **Agree** | **Strongly Agree** |
| --- | --- | --- | --- | --- | --- |
| **A** | **Prescription entry (comparison of handwritten with electronic process)** | | | | |
| **a** | 6.2 | 8.5 | 13.2 | 27.9 | 44.2 |
| **b** | 4.3 | 8.5 | 11.0 | 34.1 | 42.1 |
| **c** | 1.5 | 2.9 | 9.5 | 21.2 | 65.0 |
| **B** | **Check Prescription Information (Paper Prescription vs. Electronic Prescription)** | | | | |
| **a** | 7.0 | 9.3 | 12.4 | 30.2 | 41.1 |
| **b** | 3.0 | 3.7 | 15.2 | 31.1 | 47.0 |
| **c** | 1.5 | 2.9 | 14.6 | 38.0 | 43.1 |
| **C** | **Monitoring prescription execution intervals from the moment of prescription** | | | | |
| **a** | 4.7 | 3.1 | 10.1 | 29.5 | 52.7 |
| **b** | 2.4 | 4.9 | 15.9 | 29.9 | 47.0 |
| **c** | 4.4 | 5.8 | 16.1 | 28.5 | 45.3 |
| **D** | **Check and fill in participation rate** | | | | |
| **a** | 10.9 | 6.2 | 17.1 | 31.8 | 34.1 |
| **b** | 6.7 | 7.3 | 25.0 | 26.8 | 34.1 |
| **c** | 2.9 | 2.2 | 10.2 | 32.1 | 52.6 |
| **Q** | **Billing** | | | | |
| **a** | 7.0 | 14.7 | 23.3 | 26.4 | 28.7 |
| **b** | 12.8 | 9.1 | 31.1 | 24.4 | 22.6 |
| **c** | 1.5 | 9.5 | 18.2 | 32.8 | 38.0 |
| **E** | **Detection of possible errors in the prescription (caused by the doctor)** | | | | |
| **a** | 5.4 | 7.0 | 18.6 | 35.7 | 33.3 |
| **b** | 4.9 | 10.4 | 21.3 | 34.8 | 28.7 |
| **c** | 6.6 | 8.8 | 20.4 | 40.1 | 24.1 |
| **F** | **Actions to correct errors in the prescription (caused by the doctor)** | | | | |
| **a** | 6.2 | 5.4 | 25.6 | 34.9 | 27.9 |
| **b** | 4.9 | 13.4 | 27.4 | 28.0 | 26.2 |
| **c** | 10.9 | 15.3 | 29.2 | 29.9 | 14.6 |
| **G** | **Prescription data transfer to pharmacy data system** | | | | |
| **a** | 4.7 | 7.0 | 20.2 | 30.2 | 38.0 |
| **b** | 6.1 | 5.5 | 22.0 | 34.8 | 31.7 |
| **c** | 2.2 | 4.4 | 12.4 | 32.1 | 48.9 |
| **K** | **Provide instructions to the patients about the medicine they are taking** | | | | |
| **a** | 9.3 | 18.6 | 27.9 | 28.7 | 15.5 |
| **b** | 14.6 | 15.9 | 26.8 | 26.8 | 15.9 |
| **c** | 5.1 | 17.5 | 29.2 | 28.5 | 19.7 |
| **M** | **Signature / confirmation of receipt of medicines** | | | | |
| **a** | 14.0 | 11.6 | 22.5 | 31.0 | 20.9 |
| **b** | 11.0 | 11.0 | 27.4 | 27.4 | 23.2 |
| **c** | 20.4 | 20.4 | 20.4 | 27.0 | 11.7 |
| **T** | **Partial execution of the prescription** | | | | |
| **a** | 7.8 | 10.9 | 18.6 | 29.5 | 33.3 |
| **b** | 11.6 | 9.8 | 28.7 | 32.9 | 17.1 |
| **c** | 1.5 | 6.6 | 21.2 | 43.1 | 27.7 |
| **P** | **Prescription renewal** | | | | |
| **a** | 5.4 | 8.5 | 15.5 | 31.0 | 39.5 |
| **b** | 4.3 | 7.9 | 17.7 | 37.2 | 32.9 |
| **c** | 5.8 | 15.3 | 26.3 | 35.0 | 17.5 |
| **S** | **Delivery of requested pharmaceuticals** | | | | |
| **a** | 7.8 | 10.9 | 22.5 | 33.3 | 25.6 |
| **b** | 8.5 | 12.8 | 34.8 | 26.8 | 17.1 |
| **c** | 5.1 | 12.4 | 29.9 | 27.7 | 24.8 |

**Section B**

**Questionnaires on e-prescription (ePreQs)**

# Demographic Information (PHCPs and SMDs)

1. Gender: Male / Female / Prefer not to say

2. Age (in years):

3. Year of Graduation:

4. Medical Specialty:

5. Tenure in current unit (in years):

6. Experience using e-prescription systems (in years):

7. Do you prescribe medications? Yes / No

8. How often do you prescribe medications?

1. Daily
2. On a weekly basis
3. Less than once a week

9. Number of e-prescriptions administered per day:

1. Less than five e-prescriptions per day
2. 6-9 e-prescriptions per day
3. More than 10 e-prescriptions per day
4. More than 20 e-prescriptions per day
5. Other (please define):

# Demographic Information (Pharmacists)

1. Gender: Male / Female / Prefer not to say

2. Age (in years):

3. Year of Graduation:

4. Tenure in current unit (in years):

5. Experience using e-prescription systems (in years):

6. Do you work in a pharmacy?

1. Yes
2. No

7. How often do you process prescriptions?

1. Daily
2. On a weekly basis
3. Less than once a week

8. What percentage (%) of the prescriptions are processed through a e-prescription system?

1. < 10
2. 10–24
3. 25–49
4. 50–75
5. > 75

# Evaluation of e-prescription systems

The following sections are an adaptation of the questionnaires of [1][2][3]

Please answer the following questions by selecting the most appropriate alternative for you and / or write your answer in the space provided. It is important for the study that you answer all questions.

1. Please give an estimation of the number of e-prescriptions you administered in 2020:
2. Do you prescribe using the IDIKA system or do you use a third-party application? (If you use several systems in your clinical practice, please select the one you use more often and answer the questions about your experiences with this system).
   1. IDIKA
   2. Other (please define):
3. Has the use of e-prescriptions affected your work?
   1. No
   2. Yes (please describe how):
4. Do you think the system by IDIKA is safe from the standpoint of data protection?
   1. Yes
   2. No. (What issues do you perceive as problematic?): ........................................................
5. How often do you experience a technical problem while using the system by IDIKA that hinders / slows the administration of a prescription?
   1. Daily or almost daily
   2. About once a week
   3. A few times a month
   4. About once a month
   5. Less than once a month
   6. Never
6. For the e-prescriptions you have administered, how often have there been ambiguities or errors that have required clarification during the process?
   1. Daily or almost daily
   2. About once a week
   3. A few times a month
   4. About once a month
   5. Less than once a month
   6. Never
7. What kind of ambiguities have there been in the e-prescriptions? (please select the three most common)
   1. Incorrect pharmaceutical product
   2. No information on adverse drug reactions
   3. Incorrect pharmaceutical form (ml, mg, tabs etc)
   4. Incorrect total amount of medicine contained
   5. Unclear or incorrect dosage instructions
   6. Missing dosage instructions
   7. Useful information is missing (e.g., the patient’s weight)
   8. No information on patient’s allergies
   9. Specific dosage instructions or specific purpose of use missing
   10. Other (please define):
8. In your opinion, have e-prescriptions affected the amount of contacts between the pharmacy and physicians?
   1. No
   2. The amount of contacts have decreased.
   3. The amount of contacts have increased. What have been the main reasons for the
   4. contacts? (please elaborate)
9. Has your workplace agreed on procedures related to renewal of e-prescriptions for patients with chronic illnesses?
   1. Yes
   2. No
   3. I don’t know
10. Do you collaborate with other healthcare professionals for the renewal of e-prescriptions for patients with chronic illnesses?
    1. Yes – Please define which specialties
    2. No
    3. I don’t know
11. Do you use the option of the system to issue a referral to a doctor of another specialty or to the emergency room of a hospital?
    1. Yes
    2. No
    3. I don’t know
12. If yes, have you received an answer from the doctor / emergency room?
    1. Yes
    2. No
    3. I don’t know
13. In your opinion, are the laws and official regulations regarding e-prescription clear?
    1. Yes
    2. No (What issues do you perceive as problematic?):
14. In your opinion, what are the main benefits of e-prescription?
15. In your opinion, what are the main problems / areas needing development in e-prescription?
16. How satisfied are you with e-prescription as a whole? (Please select the most suitable alternative from 1- Not satisfied at all to 6 – Very Satisfied). (*)(***)
17. How do you proceed if / when you receive a message that the e-prescription system is not responding, given that the patient is with you and does not need the medications immediately?
    1. I submit the prescription later
    2. I print a prescription form the computer and give it to the patient
    3. I give the patient a handwritten prescription
    4. I call the pharmacy and we proceed with the prescription over the phone
    5. There is no action needed. I will administer the e-prescription once the system is again available
    6. Other (please define)
18. Proposed improvements (please select at least 3)
    1. Easy / simple drug selection
    2. Link to patients’ EHR, Medical History, Diagnoses, Comorbidities, Allergies
    3. Integration of TPPs allowing flexible interaction
    4. Communication between the prescribing clinician and the pharmacy for the availability of a specific drug
    5. Link to the Electronic Pre-Authorization System of EOPYY for prescription of off-label medication
    6. Option to issue reports, such as the list of drugs prescribed to a patient
    7. Option to cancel a prescription
    8. ABC Analysis
    9. Chat between the prescribing physician and the pharmacy
    10. Graphic representation of data
    11. Option to create and save “favorites”
    12. Reduced number of actions (mouse clicks)
    13. Improved layout
    14. Percentage of prescriptions based on medication lists with reference to originals (brand-name drugs) and generics
    15. Receipt from the pharmacy
    16. Information on drug interactions
    17. Warning for prescribing high doses (not in accordance with the drug’s SPC)
    18. Other (please define):
19. Can the system you use be link to a patient’s EHR? (**)
    1. Yes
    2. No …………………………………….
20. Are there available clinical protocols implemented in the system for the rationalization of pharmaceutical expenditure evidence based tools to support clinical decisions? (**)
    1. Yes (please define which)
    2. No ……………………………………….
21. Has your workplace written guidelines regarding error situations between the pharmacy and the e-prescription system? (***)
    1. Yes
    2. No
    3. I don’t know

**(*) PHCPs**

**(**) SMDs**

**(***) Pharmacists**

1. What is your opinion on the following statements? (Please select from 1 – Strongly Disagree to 5 – Strongly Agree)

|  | **Strongly Disagree** | **Disagree** | **Moderately Agree / Disagree** | **Agree** | **Strongly Agree** |
| --- | --- | --- | --- | --- | --- |
| The system by IDIKA is difficult to use | 1 | 2 | 3 | 4 | 5 |
| It is easy to learn how to use the system by IDIKA | 1 | 2 | 3 | 4 | 5 |
| The system by IDIKA is inflexible and I can’t use it as I would like to | 1 | 2 | 3 | 4 | 5 |
| The system by IDIKA is clear and understandable | 1 | 2 | 3 | 4 | 5 |
| The implementation of the integrated Therapeutic Prescription Protocols (TPPs) promotes good clinical practice (*)(**) | 1 | 2 | 3 | 4 | 5 |
| The implementation of the integrated TPPs is inflexible and I can’t use it as I would like to (*)(**) | 1 | 2 | 3 | 4 | 5 |
| It is easy to learn how to use the integrated TPPs in the system by IDIKA (*)(**) | 1 | 2 | 3 | 4 | 5 |
| It is necessary to link the e-prescribing system to the patients’ EHR, Medical History | 1 | 2 | 3 | 4 | 5 |
| Registration in the system of all administered drugs (positive list, non-prescription drugs) is useful (***) | 1 | 2 | 3 | 4 | 5 |

1. In your opinion, how have e-prescriptions affected the various phases of prescribing a medication? (Please select from 1 – Strongly Disagree to 5 – Strongly Agree)

| **[e-Prescription has facilitated…]** | **Strongly Disagree** | **Disagree** | **Moderately Agree / Disagree** | **Agree** | **Strongly Agree** |
| --- | --- | --- | --- | --- | --- |
| Prescription entry (comparison of handwritten with electronic process) | 1 | 2 | 3 | 4 | 5 |
| Check Prescription Information (Paper Prescription vs. Electronic Prescription) | 1 | 2 | 3 | 4 | 5 |
| Monitoring prescription execution intervals from the moment of prescription | 1 | 2 | 3 | 4 | 5 |
| Check and fill in participation rate | 1 | 2 | 3 | 4 | 5 |
| Billing | 1 | 2 | 3 | 4 | 5 |
| Detection of possible errors in the prescription (caused by the doctor) | 1 | 2 | 3 | 4 | 5 |
| Actions to correct errors in the prescription (caused by the doctor) | 1 | 2 | 3 | 4 | 5 |
| Prescription data transfer to pharmacy data system | 1 | 2 | 3 | 4 | 5 |
| Provide instructions to the patients about the medicine they are taking | 1 | 2 | 3 | 4 | 5 |
| Signature / confirmation of receipt of medicines | 1 | 2 | 3 | 4 | 5 |
| Partial execution of the prescription | 1 | 2 | 3 | 4 | 5 |
| Prescription renewal | 1 | 2 | 3 | 4 | 5 |
| Delivery of requested pharmaceuticals | 1 | 2 | 3 | 4 | 5 |

1. What is your opinion on the following statements? (Please select from 1 – Strongly Disagree to 5 – Strongly Agree)

|  | **Strongly Disagree** | **Disagree** | **Moderately Agree / Disagree** | **Agree** | **Strongly Agree** |
| --- | --- | --- | --- | --- | --- |
| e-Prescription reduces the risk of errors | 1 | 2 | 3 | 4 | 5 |
| e-Prescription promotes better management of the patients overall medication when administering a drug | 1 | 2 | 3 | 4 | 5 |
| e-Prescription does not facilitate the monitoring of multiple drug therapy | 1 | 2 | 3 | 4 | 5 |
| e-Prescription does not promote monitoring of adverse drug reactions / side effects | 1 | 2 | 3 | 4 | 5 |
| e-Prescription facilitates the monitoring of drug to drug interactions | 1 | 2 | 3 | 4 | 5 |
| e-Prescription does not lessen the risk of incorrect interpretation of a prescription at the pharmacy (compared to a handwritten prescription) | 1 | 2 | 3 | 4 | 5 |
| e-Prescription lowers the number of prescription forgeries | 1 | 2 | 3 | 4 | 5 |
| For the patients there is no difference between e-prescription and handwritten prescription | 1 | 2 | 3 | 4 | 5 |
| The patients receive sufficient information about their e-prescription before visiting the pharmacy | 1 | 2 | 3 | 4 | 5 |
| When the patients arrive at the pharmacy they usually have instructions with them regarding their e-prescription | 1 | 2 | 3 | 4 | 5 |
| ePrescription has made it easier to provide customers with up-to-date information about their prescriptions and the medications | 1 | 2 | 3 | 4 | 5 |
| Physicians actively use the e-prescription system’s message space to communicate information between the physician, the pharmacy, and the patient | 1 | 2 | 3 | 4 | 5 |
| The patients find it difficult to determine the medication amount received from the e-prescription | 1 | 2 | 3 | 4 | 5 |
| The patients need more information from the pharmacy’s staff about their e-prescription when they visit the pharmacy | 1 | 2 | 3 | 4 | 5 |

**References**

1. Kauppinen, H., Ahonen, R., & Timonen, J. (2017). The impact of electronic prescriptions on the medicine dispensing process in Finnish community pharmacies – a survey of pharmacists. In *Journal of Pharmaceutical Health Services Research* (Vol. 8, Issue 3, pp. 169–176). https://doi.org/10.1111/jphs.12185
2. Kivekäs, E., Enlund, H., Borycki, E., & Saranto, K. (2016). General practitioners’ attitudes towards electronic prescribing and the use of the national prescription centre. In *Journal of Evaluation in Clinical Practice* (Vol. 22, Issue 5, pp. 816–825). https://doi.org/10.1111/jep.12548
3. Hellström, L., Waern, K., Montelius, E., Åstrand, B., Rydberg, T., & Petersson, G. (2009). Physicians’ attitudes towards ePrescribing – evaluation of a Swedish full-scale implementation. *BMC Medical Informatics and Decision Making*, *9*(1), 37. https://doi.org/10.1186/1472-6947-9-37
